# A systematic-narrative hybrid review of evidence: Exploring how corporate social responsibility initiatives impact population health

**DOI:** 10.1177/13634593241313433

**Published:** 2025-01-18

**Authors:** Toby Freeman, Kristen Foley, Julia Anaf, Beth Nosworthy, Fran Baum

**Affiliations:** University of Adelaide, Australia; University of Adelaide, Australia; University of Adelaide, Australia; University of Adelaide, Australia; University of Adelaide, Australia

**Keywords:** commercial determinants of health, corporate social responsibility, government regulation, health equity, population health, power relationships

## Abstract

Corporate Social Responsibility (CSR) refers to initiatives undertaken by corporations that aim to make a positive impact on society. It is unclear to what extent these aims are achieved in relation to population health. We explored the evidence for mechanisms by which CSR has positive or negative effects on population health through a systematic-narrative hybrid review of 97 relevant articles. We found few examples overall that could trace a CSR initiative through to verifiable impacts on the population. Our review found that generally the evidence for the impacts of CSR on population health was patchy, highly heterogenous and of varying quality. We found some potential positive impacts of CSR on health; including on poverty alleviation, development, health care, the environment and the health and wellbeing of workers. Some CSR initiatives were rebranding of core functions, such as HR practices and employee wellbeing strategies, or were a partial redressing of the problems the corporation itself is creating, such as CSR initiatives that sought to improve workplace safety, reduce corporate environmental footprints or relocate people displaced by mining activities. We situate these impacts in relation to the role and intent of CSR, and argue that meaningful progress on CSR can only be made with greater transparency and reporting of initiatives to more fulsomely evaluate their impacts – as well as the political economy in which these sit. It is further critical to strengthen government regulation and oversight to maximise any public good that can come from CSR, and minimise the negative consequences reported in research literature.

## Introduction

This paper focuses on Corporate Social Responsibility as an element of the ‘commercial determinants of health’ – exploring how corporations, and the profit motive, as well as the structures that support and prioritise commercial interests, influence population health (Gilmore et al., 2023; Glover and Petticrew, 2021; Lee and Freudenberg, 2022; Maani et al., 2020). Research in this field has examined health impacts of harmful commodities (e.g. tobacco, alcohol, ultraprocessed foods, Freudenberg et al., 2021), global trade agreements (Friel et al., 2015) and the operations of transnational corporations (Baum et al., 2016). The ways in which commercial processes affect health are vast, with four industries (tobacco, alcohol, ultra-processed food and fossil fuels) estimated to be responsible for over a third of deaths worldwide each year (Gilmore et al., 2023). Many transnational corporations have wealth that far exceeds many nations, guarded and grown through broad and easy access to global and national decision making institutions and processes (Baum and Anaf, 2015; de Lacy-Vawdon et al., 2022; Gilmore et al., 2023; McKee and Stuckler, 2018).

Less evidence addresses positive influences that commercial sectors can have on population health, and the mechanisms for these influences are less clear. Employment is mainly a positive determinant of health (Noordt et al., 2014), as long as it is a decent job (Butterworth et al., 2011). Corporations may also pay taxes which become public revenue to support population health and social determinants of health. Many corporations develop Corporate Social Responsibility (CSR) initiatives with the specific intention to affect society positively. CSR stems from the belief that corporations have responsibilities to better society beyond just generating profit for shareholders, which can be economic, legal, ethical and/or philanthropic (Carroll and Shabana, 2010). Related concepts include corporate sustainability and corporate citizenship. Sheehy and Farneti (2021) argue that CSR is distinct from these other terms as it is a ‘bottom-up, organisation-driven idea’ (p. 6) whereas terms such as corporate sustainability are imposed from global policy agendas. CSR highlights the role of organisational policy and behaviour, and responsibility for social, employee, stakeholder and environmental issues (Sheehy and Farneti, 2021). Sheehy (2015) defines CSR as:

a socio-political movement which generates private self-regulatory initiatives, incorporating public and private international law norms seeking to ameliorate and mitigate the social harms of and to promote public good by industrial organisations. (p. 639)

There has been both scepticism and criticism of the concept of CSR. Scholars of commercial determinants of health have raised concern about the extent of actual positive impacts of CSR, arguing that ‘evidence indicates that it is at best a superficial, public relations exercise’ (Gilmore et al., 2023: 1202) and a way to influence policy. Conversely, Milton Friedman argued in his newspaper article ‘The Social Responsibility of Business is to Increase its Profits’ that:

The businessmen believe that they are defending free enterprise when they declaim that business is not concerned “merely” with profit but also with promoting desirable “social” ends. . . In fact they are . . . preaching pure and unadulterated socialism. (Friedman, 1970: 17)

Rendtorff (2011) charted the global rise of CSR, and cited as key drivers the European Union movement for sustainable development, and in the US, the Federal Sentencing Guidelines for Organisations in 1991, which indicated a positive reputation for CSR can reduce sentences for corporate wrongdoing. Rendtorff (2011, 2020) argued that CSR enables corporations to fulfil the function of gaining social legitimacy. Political Corporate Social Responsibility (Scherer and Palazzo, 2011) identifies that corporations can also be powerful political actors – a challenge that is under-researched in CSR literature. In some cases of large corporations, political CSR can involve the corporation assuming an almost ‘a state-like role’ (Scherer and Palazzo, 2011: 900), taking on some of the responsibilities and functions usually accorded to governments. Corporations exercise their significant financial and cultural power to engage in political issues to profit ‘from economic windfalls of right-wing economic policy, while also benefiting from the cultural legitimacy afforded by being associated with ‘good’ progressive causes’ (Rhodes, 2021: 43).

Little is known about its impacts of CSR on population health via its possible influence on a range of social determinants of health, including environmental health, working conditions and community development. Our manuscript seeks to develop a modest exploratory starting point for this work, by reviewing existing research to understand:

What is the evidence for mechanisms by which Corporate Social Responsibility has positive or negative effects on population health?

The evaluation of CSR initiatives is necessarily complex, in part because of the heterogeneity of CSR initiatives, because they seek to act on broad and diverse drivers of health; because they take place in diverse political and cultural contexts; because they can unfold over varying lengths of time; utilise different measurement techniques; and are published about in grey and peer-reviewed literature. Despite the difficulty of undertaking such work, its value in injecting evidence about ‘real’ impacts of CSR on health is important to further our understanding of commercial determinants of health – and to contextualise the reality of positive intentions which CSR aims to achieve.

## Methods

We used a systematic-narrative hybrid review (Turnbull et al., 2023). Narrative reviews rely on scholarly summary via interpretation and critique (MacLure, 2005) to apply expert knowledge to research problems in ways which generate academic insight (Greenhalgh et al., 2018). While narrative reviews are sometimes criticised for potential bias in the selection of included literature, systematic-narrative hybrid reviews meld narrative elements with the strengths of a stringent and reproducible search approach (Turnbull et al., 2023). Reliability and validity are pursued through consideration of: (1) a research question, (2) justification, (3) literature sources, (4) search parameters, (5) data cleaning and (6) information synthesis (Turnbull et al., 2023). The search protocols and inclusion/exclusion criteria draw from systematic review practices and include multidisciplinary databases, reducing the extent to which the review could be ‘biased’ by only drawing on certain literatures (Greenhalgh et al., 2018).

### Search design

**Figure 1.**
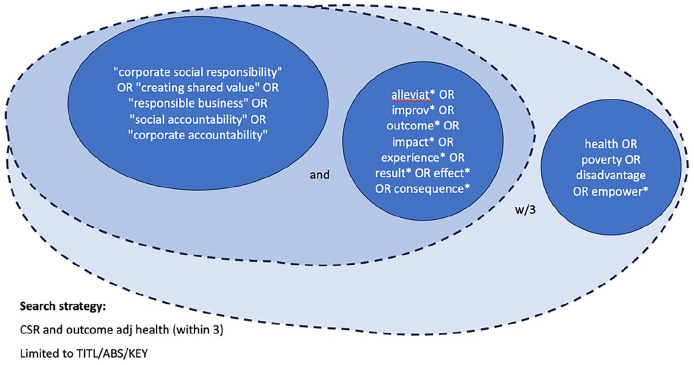
Search logic, terms and operators used.

The review was guided by our multidisciplinary team (public health, sociology and law) with advice from a university librarian. Initial search terms were trialled in SCOPUS (September 2022) with a high return rate (>24,000) but low utility/relevance (~1/20 on hand review of titles/abstracts). A major confounding factor was that most articles lacked evidential support. We removed less relevant terms (‘sustainability’, ‘stakeholder management’ and ‘philanthropy’), added proximity indicators to strengthen the link between health and outcomes (which improved relevance to 5–6/10); and added empowerment, poverty and disadvantage to return results which more clearly reflected articles we were seeking. Finalised search strings/terms are shown in Figure 1.

Scopus, Web of Science and Proquest were selected for inclusion of broad peer-reviewed literature relevant to the research question. Business Source was also included because it indexes SSRN, a database highly relevant to legal aspects. Lastly, we searched Google Scholar (with a simpler search string), because while there is no transparency about the algorithm used (and searches therefore can’t be replicated), it can yield highly relevant articles and thus be a useful complement to other more rigorous search strategies. The first 20 pages of Google Scholar results were reviewed, after which relevance dropped off. For all databases results were date limited (2009–onwards), reflecting the proliferation of CSR initiatives and terminology.

### Search results

Searches were run in November 2022. A total of 494 unique records were returned. These were allocated randomly to the authors, so that each author reviewed 50–100 articles, screening the title and abstract for suggested inclusion or exclusion (judged per whether the work contained evidence pertinent to the research question). These decisions were reviewed by the full team. The resulting 151 articles were then categorised into broad categories (e.g. food, poverty/development, governance and regulation and health and social services) and categories were assigned to authors based on their expertise to do a full text review and summary for each category. During this stage, a further 54 articles were removed as not relevant to the research question after full text review, leaving a pool of 97 included articles (see Table 1).

**Table 1.** Database search results, screening outcomes and final number of articles included in the review.

Database	Returns
Google scholar	150
SCOPUS	234
Web of science	188
ProQuest	382
Business source complete	149
Total retrieved articles	1103
De-duplicating removed	609
Remaining articles for inclusion in abstract screening	494
Articles excluding by authors during abstract screen	343
Articles excluded after full text reviewed	54
Final number of articles reviewed	97

Each author reviewed and synthesised the literature within their chosen categories, producing a written summary that was discussed with the full research team and explored in relation to findings from other categories and the full picture of evidence developing. During collaborative workshops we identified key themes and findings based which were further debated and refined during manuscript development.

## Results

Below we present an overview of the literature; comprised of (1) types of evidence returned for consideration; and (2) the differing conceptions of CSR present.

We then present the evidence for (3) positive, and (4) negative and null impacts of CSR on health. Through this we identified and have reported on (5) power relationships, and (6) government regulation of CSR.

### Overview of the literature

Here we showcase key features of retrieved literature.

#### Types of evidence for consideration

Our search returned diverse evidence ‘types’, drawn from around the world, and from different disciplines (see Table 2). Geography was determined by the source of the empirical data used or cited in the article. Discipline was determined from the journal field, using Australian Bureau of Statistics Field of Research Divisions (Australian Bureau of Statistics, 2020). For references other than journal articles, the content was reviewed to determine the discipline.

**Table 2.** Types of evidence returned, geography the research focused on, and the disciple of study (*N* = 97 references).

Characteristic	Number (%)
Type of evidence	
Empirical evidence of impacts	29 (30%)
Evidence on perceptions of impact^a^	29 (30%)
Opinion piece, commentary or editorial citing evidence	20 (21%)
Analysis of non-academic documents^b^	12 (12%)
Review of academic literature	7 (7%)
Geography^c^	
Asia	33 (34%)
Africa	21 (21%)
Global	14 (14%)
Europe	8 (8%)
USA	7 (7%)
Middle east	5 (5%)
Australia	4 (4%)
UK	2 (2%)
Discipline	
Commerce, management, tourism and services	54 (56%)
Medical and health sciences	20 (21%)
Studies in human society	9 (9%)
Environmental sciences	7 (7%)
Earth sciences	2 (2%)
Built environment and design	1 (1%)
Education	1 (1%)
Engineering	1 (1%)
Psychology and cognitive sciences	1 (1%)
Law and legal studies	1 (1%)

a Examples of articles coded as ‘evidence on perceptions of impact’ included community surveys seeking their evaluation of CSR impact.

b Examples of analysis of non-academic documents were studies reviewing corporate policies and reports to analyse CSR.

c Geography sums to 98 because one article compared data from the USA and Korea.

One reason for the volume of Asian literature may be that some Asian countries have mandated CSR. In Indonesia, all corporations ‘exploiting natural resources’, and all ‘capital ventures’ need to spend a percentage of their profits to charity or CSR (Rela et al., 2020). India has mandated that corporations meeting net profit, worth and/or turnover criteria ‘have to spend 2% of their net profits on social development or explain the reasons for not spending through their annual report disclosures’ (Ghosh et al., 2021: 90).

The literature included different disciplinary positions and theoretical orientations, which adds another layer of heterogeneity (Table 2). These positionalities are important because the extent and nature of the available evidence reflects the disciplines which have developed knowledge about CSR. We found the extent of critical interrogation of corporate activities varied substantially, from articles that took a very sceptical stance, through to articles which accepted corporate claims at face value. We took into account the strength of the evidence for the claims made. These key features of the literature underpin what sense can be made from evidence about CSR and impacts on health.

#### Different conceptions of CSR

We encountered different definitions and theoretical frameworks for CSR, echoing that no agreed definition exists (Xiong and Luo, 2021) and justifying narrative approaches for evidence-development. Generally, definitions described CSR as comprising actions by corporations to contribute to society outside of profit-generation alone. This variation may partly reflect different cultural contexts. Uduji et al. (2018) urge that CSR should be considered differently in low and middle income countries than in high income countries; as the social, economic and cultural contexts differ and governments in low and middle income countries tend to lack resources for development, power to pressure transnational corporations, and at times, political will to do so.

Frameworks have been proposed to categorise CSR activities. One widely cited and useful categorisation is Carroll’s Pyramid of CSR (Carroll, 1991), which identifies four areas of CSR starting from the bottom up: economic (the responsibility to maintain profitability), legal (ensuring operations and practices abide by laws and regulations), ethical (ensuring operations and practices are consistent with human rights, and the good of individuals, society and the natural environment) and philanthropic (contributing voluntarily to the wellbeing of people and society).

As Carroll’s (1991) pyramid suggests, CSR can be focused on internal matters (i.e. the day-to-day operations and practices of a company, and whether or not these are harmful) or external (in terms of philanthropy aimed at the broader community), and can focus on people, the environment or the economy, in terms of community development or poverty alleviation. We saw all of these elements as potentially relevant to health, since natural environment and economic factors including poverty and development are key determinants of population health (Commission on the Social Determinants of Health, 2008). Thus, we examined the literature for evidence of mechanisms leading to positive or negative effects on health in all these categories.

### Evidence of impacts of CSR on health

#### Evidence for positive impacts

We found evidence for positive impacts of CSR activities on people’s health in relation to: poverty alleviation and development; environmental impact; health and wellbeing of employees and health care.

##### Poverty alleviation and development

Studies from Indonesia were identified that found positive results of CSR on poverty alleviation and development. Rela et al. (2020) surveyed a community in Indonesia that had been in receipt of a CSR initiative from the nickel mining industry. They found that community members reported improved social, economic and environmental wellbeing because of the CSR. An evaluation of an Indonesian CSR programme focusing on loans, training and business development improved public welfare through supporting community businesses (Soedarsa, 2019), and an analysis of the CSR of a cement company found improved resources for the community, including health care, education and business opportunities (Rustinsyah, 2016).

Chauhan and Sukhmani’s (2020) overview of the CSR projects that have been implemented in India found CSR initiatives had contributed to community development through support for agribusiness and rural economic development programmes. While Wuttke and Vilks’ (2014) review of CSR by Indian construction companies found only 2 out of 10 met their criteria for good practice, they did find some positive examples of development activities, such as water supply projects, vocational training centres and a school for the children of construction workers. In Nigeria, Amadi and Abdullah (2012) reported on Shell Petroleum Developing Company’s CSR efforts to alleviate poverty through community education and opportunities for youth development. Positive impacts of CSR included that the company established and funded health services and health initiatives including anti-malaria, HIV/AIDS treatment and immunisation programmes, sponsored rural school teachers and trained and employed local youths. Wadvalla (2016)’s study documented how two large global companies, IKEA and Ericsson, took action to minimise their use of cheaper labour in supply chains. Both companies saw improving skills in their supply chain staff as a form of CSR. This approach included IKEA’s goal to ‘Help lift people out of poverty by providing good places to work throughout our value chain’ (Wadvalla, 2016: 24), aiming to ensure good working conditions at all skill levels while also preventing child labour in their supply chain.

##### Environmental impact

Healthy natural environments are critical determinants of good health yet are severely under threat through pollution, deforestation, climate change and degradation, chiefly driven through for-profit actor practices (Kinda and Thiombiano, 2021). Conservation work and minimising/compensating for negative impacts were cornerstone CSR strategies in this area; with IKEA reported to have invested in projects to lower the environmental footprint of their supply chain, partnering with NGOs such as Greenpeace, Save the Children and WWF (Wadvalla, 2016). Other examples of environmental CSR included tree planting, and promoting renewable energy (Chauhan and Sukhmani, 2020; Junior et al., 2022; Rustinsyah, 2016).

##### Health and wellbeing of employees

CSR was argued to improve employee health and wellbeing through a number of strategies. Firstly, many articles framed employment as a positive outcome in itself, in some cases as a charitable output by corporations (Rela et al., 2020; e.g. Panigrahi and Sheela, 2015). Similarly, Medina et al. (2020) classed the voluntary payment of a living wage (compensation for workers that allow them a decent quality of life for themselves and their dependents) as a form of CSR.

Secondly, occupational health and safety initiatives were often included under CSR (AlFadhli, 2019; Montero et al., 2009; Pearse, 2010), particularly for workers in supply chains in low income countries (Brown, 2007). Some authors argued CSR could motivate corporations to improve occupational health and safety where legislation and other protections have failed to ensure adequate workplace safety (Montero et al., 2009; Oliinyk, 2017). A study of Korean companies found that better CSR performance (as scored by the Korea Corporate Governance Service) was associated with less workplace injury, suggesting that more socially responsible companies tend to invest more in workplace safety (Koo and Eun Sun, 2020).

Thirdly, companies’ CSR was seen to demonstrate positive corporate values and signal to workers that it was a caring employer (Calnan, 2015; Mao et al., 2021; Papasolomou, 2017; Roman-Calderon et al., 2015; Stankeviciute and Wereda, 2020). One example was companies that allowed employees to volunteer for causes during their employed time (Calnan, 2015). Positive effects of CSR on worker psychological wellbeing was a consistent finding, with employee surveys finding CSR was associated with increased organisational trust and psychological empowerment (Memon et al., 2021), satisfaction with the company’s COVID-19 response, and hope, optimism, resilience and self-efficacy (Mao et al., 2021), happiness at work (Jae-Geum et al., 2022a, 2022b), work satisfaction (Zink, 2014), employee engagement (Mei Peng and Spong, 2022), and occupational wellbeing (Stankeviciute and Wereda, 2020). A survey of Australian employees found similar results but noted that CSR did not explain variance in employee engagement over and above traditional HR practices (Smith and Langford, 2011), suggesting CSR may influence employees’ engagement through fostering positive HR practices.

##### Health care

Positive evidence of CSR initiatives included provision of health care services and hospital facilities and training of healthcare workers (Adjei et al., 2022; Amadi and Abdullah, 2012; Rustinsyah, 2016). CSR also supported healthcare improvements including provision of palliative care, vaccinations, disease research and access to healthcare for company workers (Choudhary and Singh, 2020; Morgado, 2021). Evaluation of a CSR-driven football participation programme to improve mental health in the UK (Henderson et al., 2014) found no improvement in participants’ mental health or social resources, although potential improvements in social capital through improving participants’ access to people with a particular skill in their social network; with focus group data outlining the programme’s perceived usefulness to participants in terms of social interaction and self-confidence. Kulkarni et al. (2017) argued in a commentary how CSR can be used to improve consumer safety of dietary supplements sold for weight loss and muscle building, though the authors present an uncritical view of involving corporations to promote consumer safety, that is, ‘advocacy campaigns must be open to inviting supplement manufacturers and retailers to the discussion table’ (Kulkarni et al., 2017: 95).

#### Evidence of negative or null impacts of CSR on health

The reviewed articles provided evidence for negative CSR impacts on population health and wellbeing, in terms of its use by health-harming corporations, by co-opting existing initiatives, and through having negative consequences for workplace safety.

##### CSR by health-harming corporations

CSR initiatives undertaken by tobacco companies were viewed critically; with Kostygina et al. (2022) and Friedman (2009) arguing these companies intentionally pursued CSR on underage smoking and vaping using methods they knew were ineffective in reducing use, solely to boost public relations. Friedman (2009) described this CSR as ‘shield and sword’ (a shield to protect reputation and a sword to fight off litigation/regulation) approach to reduce likelihood of public health regulation. Since regulation is effective at reducing smoking and vaping rates – and their health consequences (Bhalerao et al., 2019) – attempts by tobacco companies to supplant regulation with voluntary-yet-ineffective CSR has a net negative effect on population health. Friedman highlights a deeper, fundamental impossibility of health-harming companies practising CSR, in this case tobacco corporations, that: ‘the tobacco industry’s products are lethal when used as directed, and no amount of public relations or funding of ineffective youth smoking prevention programmes can reconcile that fundamental contradiction with ethical corporate citizenship’ (Friedman, 2009: 819).

CSR undertaken by extractive industries were identified to have negative impacts in their own right, in addition to the health-harming effects of their broader corporate activity. Baumuller et al. (2011) investigated CSR by oil companies in the Niger Delta and found very mixed results, indicating the CSR initiatives ‘were found to create greater divisions and inequalities in and between communities’ (p. 33), and that communities did not have meaningful ownership of the initiatives. The report quotes the UN saying oil companies’ CSR efforts were ‘piecemeal and short-term’ (p. 48). Uduji and colleagues similarly found mining company CSR in Nigeria had weak benefits, and exacerbated age and rural/urban inequities (Uduji et al., 2018, 2021). A Zimbabwean mining company’s CSR was critiqued for not involving the community enough, being largely limited to ‘erratic food donations’, with a well built for the village becoming contaminated by toxins from mining (Chimeri, 2016). Chimeri (2016) argued these CSR efforts were an exercise in minimal philanthropy to gain a social licence to maximise profits, citing the support for relocation of only 100 of 600 families displaced due to mining activity; in doing so avoiding their legal responsibility to the other 500 displaced families and being able to label the relocation support they did provide as philanthropic CSR. These CSR outcomes are then not only negative but have obfuscatory and distracting purposes.

Another Zimbabwean case study found similarly only a fraction of households displaced by mining companies were relocated, while environmental contamination from the mining was rife (Nhavira, 2019). Surveys of six Ghanian communities involved with mining company CSR were surveyed and reviewed the CSR initiatives negatively, with 77% reporting that poverty, cost of living, crime and environmental degradation all increased alongside corporate activity (Shubita et al., 2023).

##### Co-option of existing initiatives

Dolan (2010) provided an example of the co-option of a CSR-type initiative, Fairtrade, once based on social justice, equity and community solidarity; but now subject to the power of global food corporations which ‘impoverished [Fairtrade’s] capacity to deliver empowerment, autonomy and economic justice’ (p. 41).

##### Workplace safety

While we did find some positive evidence of CSR on employee health and wellbeing (summarised above), Brown (2007) argued that corporations’ health and safety CSR codes of conduct have only seen very marginal improvements in working conditions in supply chains of low income countries, and are largely public relations exercises with no funding or follow through. This was demonstrated in a case study of a footwear factory that supplies Reebok, which had improved their codes of conduct, and succeeded in reducing the most egregious working conditions such as child labour and corporal punishment of workers. While Reebok gained the social benefits of these actions as the last part of the supply chain, they did not contribute materially to implementing better labour standards; and because of the need to keep competitive in the labour market, the factory ended up cutting wages and increasing workloads. Pearse (2010) argued that shortcomings in CSR reporting for occupational health and safety (see also AlFadhli, 2019), including lack of standardised reporting, lack of detail and lack of external auditing, constrain the potential for CSR to improve occupational health and safety effectively.

#### Power and CSR

A number of articles raised concerns with power relationships in CSR. Chaudhuri and Morash (2019) note that CSR allows corporations to set their own development agenda. For example, a review of pharmaceutical firm CSR in Brazil found that CSR initiatives were not well aligned with government priorities around enhancing medicine and health care access (Thorsteinsdóttir et al., 2017). One strategy to counter this agenda-setting power was the Global Memorandum of Understanding (GMoU), used in Nigeria for multinational oil company CSR efforts (Uduji et al., 2022). A GMoU is a written statement between the oil companies and communities. The idea of GMoUs is that communities can decide the development they want, and the companies provide funding. While this sounds like positive change for the communities, the authors reported mixed findings for the GMoU model, with some reports claiming they have undermined human development and others reporting limited success (Uduji et al., 2022). Uduji’s own work suggested that GMoUs had been successful in improving women’s education attainment and reducing violence against women (Uduji et al., 2022), but not successful in reducing rates of HIV/AIDS (Uduji et al., 2019) or job creation amongst youth (Uduji et al., 2021)

Ruppen and Brugger (2022) argued that mining company CSR initiatives are ‘rarely effective’ (p. 1) in part because of the imbalance of power between the actors. They argue for a political economy analysis of CSR that can take into account ‘the power dynamics underlying struggles over natural resources and shows how race, class and gender structures create inequality, marginalisation and injustice in access to and use of resources’ (Ruppen and Brugger, 2022: 3). Garvey and Newell (2005) similarly argue that voluntary philanthropic CSR ensures the power remains with the corporations, not the communities affected, and this CSR needs to be balanced against the power relationships that the CSR is masking. They give the example that the ‘huge rents that Nigerian military governments have received over a number of decades from Shell’s operations in the Niger Delta, for example, served to strengthen government resolve to silence local activists campaigning against the environmental and social impacts of oil extraction’ (Garvey and Newell, 2005: 393). Shubita et al. (2023) also note that ‘In 1995, Shell was accused of being complicit in the execution of activists in Nigeria. To rebuild its reputation, Shell started producing CSR reports and undertaking various initiatives’.

A clear thread in the more critical accounts of CSR was that while the positive impacts of CSR were nebulous, the negative impacts were difficult to trace, and the benefits to the corporations were multifaceted. CSR was seen as a way of managing reputation, avoiding regulation and legitimising the practices of health harming corporations (Fooks et al., 2013; Panigrahi and Sheela, 2015). The desired effect of CSR was often perceived to be to persuade communities to allow the corporation to conduct their business operations without interference, despite the negative impacts of that business on communities and the environment (Chaudhuri and Morash, 2019; Panigrahi and Sheela, 2015).

The previously cited benefits of CSR for employees also blur into benefits for the corporations, as these positive effects for employees also lead to reduced turnover, greater organisational commitment and motivation, greater productivity and extra-role contributions (Jae-Geum et al., 2022a; Mei Peng and Spong, 2022; Memon et al., 2021; Zink, 2014). Publicity around CSR on employee issues such as safety also improves company reputation (Koo and Eun Sun, 2020); a benefit that circulates back to companies.

#### Need for government regulation

Many authors were clear that CSR is often pursued to fend off regulation, and that regulation of corporate practices was critical to safeguard population health. The need for regulation is particularly salient for developing countries that provide transnational corporation supply chains (Yu, 2008). Such regulation may help safeguard health through improving workplace safety, worker wellbeing and reducing health-harming environmental degradation to a greater and more comprehensive degree than voluntary CSR (see e.g. Chimeri, 2016; Brown, 2007). Sjåfjell and Taylor (2019) promote a ‘regulatory ecology’ of corporate purpose, interacting with law and social norms, within the market architecture, while acknowledging that the prevailing social norm is the prioritisation of profit maximisation for shareholders and the externalisation of social and environmental costs:

This places legal compliance with social and environmental standards in a tension with perceived legal obligations under company law. This can quickly become a serious risk of non-compliance where deterrence is weak, that is where social and environmental regulation is not consistently and effectively enforced. (p. 54)

Many jurisdictions remain shareholder-primacy focused, which, at its most basic, ascribes director responsibility to act in the best interests of the company as a duty to maximise the return to the shareholders (Lipton, 2018), although pressure (including from citizens) to pursue purposes beyond shareholder profit is growing (Roe, 2021).

There is a need to regulate for more comprehensively documented CSR outcomes, so that the impacts can be better ascertained (AlFadhli, 2019). Currently, the lack of standardised reporting limits our knowledge of CSR’s actual outcomes, especially in low and middle income countries (AlFadhli, 2019; Blowfield, 2007).

## Discussion

In our exploration of evidence for the health impacts of CSR, we found that generally the evidence for the impacts of CSR was patchy, highly heterogenous and of varying levels of quality, with few examples that could trace CSR initiatives through to verifiable impacts on the population (whether these be positive or negative). CSR itself covers a vast array of initiatives, with the core defining feature being the initiation and control of the initiative by a corporate actor. Nevertheless, the literature indicates the potential for positive impacts of CSR on poverty alleviation, on reducing the negative environmental consequences of corporate practices (even if these are paradoxically caused by the same corporation), and on the health and wellbeing of the employees of the corporations engaging in CSR, all of which may improve population health.

However, we encountered an array of caveats to these potential benefits which makes it very difficult to provide a general endorsement of CSR as a strategy for improving population health. Firstly, CSR serves functions of improving public image and avoiding regulation (Friedman, 2009). For health harming corporations then, the net effect of CSR can very well be expected to be negative, as it risks perpetuating and masking the ongoing harms to health the corporation is causing. This is most clearly seen for tobacco companies (Friedman, 2009; Kostygina et al., 2022), and in the local environmental CSR efforts of extractive companies that still provide fossil fuels to the global market.

Secondly, some CSR initiatives were rebranding of core functions, such as HR practices and employee wellbeing strategies, or were a partial redressing of the problems the corporation itself is creating, such as CSR initiatives that sought to improve workplace safety, reduce corporate environmental footprints or relocate people displaced by mining activities. Highlighting such activities as CSR maximises the positive image benefits of the work, which may not be accompanied by any change in practice, or may only partially offset the harms for which the corporation is already responsible. These latter instances highlight the importance of regulation to limit corporations’ cost externalisation, by holding corporations financially accountable for the harms their practices or products cause (Wood et al., 2023).

Thirdly, CSR places corporations in the driver’s seat, addressing issues corporations have chosen, rather than democratically elected governments, or communities themselves; and with no recourse to equity or evidence. The negative consequences of this can be seen throughout our findings including that: CSR was not found to address what the government saw as priorities in Brazil; that corporations wielded considerable power, creating unequal power relationships in any community participation in CSR initiatives; that CSR bolstered corporate power to influence government; and that CSR can have adverse impacts on their supply chain, and on communities, when enacted poorly. The potential for CSR to alleviate poverty is a frequent argument in pro-CSR literature (Merino and Valor, 2011). While we found some positive impacts of CSR on poverty alleviation, the extent of the negative impacts evident in research findings indicates that leaving such vital human development goals in the hands of corporate philanthropy is a very fraught solution. Rather, supporting universal human rights to health and wellbeing, and decent living and working conditions (Braveman and Gruskin, 2003), and strategies such as strong, progressive taxation systems to ensure government resources, would be a much more reliable, transparent, and beneficial foundation on which to eradicate poverty. There is the risk that CSR ‘may distract political and social agents from the task of advancing good governance systems’ (Merino and Valor, 2011: 160). While studies identified in our search found some positives for government-mandated CSR, such as in Indonesia, other studies have found more mixed results, highlighting examples of fraud and corruption (Sheehy and Damayanti, 2019).

These findings highlight why it is important to consider the potential health impacts of CSR within the context of the global political economy from whose structure and operation corporations currently benefit considerably (Gilmore et al., 2023). The frameworks and definitions for commercial determinants of health demonstrate the ways in which these structures do this (Freudenberg et al., 2021; Gilmore et al., 2023). Our mixed findings on CSR, together with the way CSR may serve the profit-making interests of corporations, indicate that governments, international organisations and civil society play a critical role in negotiating the future business legitimacy of corporations (Rendtorff, 2019, 2020). Carroll (2015) argues that a growing emphasis on CSR in the 1970s followed a period of civil society activism on consumer rights, women’s rights and environmental protection, some high profile corporate failures of responsibility, and increasing governmental scrutiny of corporate practices. Subsequently, neoliberal approaches to public policy and to global institutions since the 1980s, promulgated in no small part by corporate interests, have seen a reduction in the role of governments, and an increase in privatisation of public assets and services (Gilmore et al., 2023; Hodge, 2018). For example, in Australia, privatisation has seen government-owned enterprises dropping from 7% of Australia’s gross domestic product in 1989–1990, to 1.3% in 2011–2012 (Abbott and Cohen, 2014). The reduced vision for the role of government includes less government intervention in markets, which has meant less regulation and less oversight and safeguarding of the private sector’s influence on population health (Gilmore et al., 2023). The reduced vision and role of government under a global neoliberal policy environment leaves a void into which corporate actors step to utilise varying CSR strategies and advance the profit motive. Along with Uduji et al. (2018), there is a growing literature highlighting the importance of attending to the differences in the conception and implementation of CSR in developing countries and developed countries, including differences in local histories and contexts and the salience of different actors (Jamali and Carroll, 2017; Jamali and Karam, 2018). Our findings show it is vital to attend to the often unbalanced power relationships between wealthy transnational corporations, the global institutions that support them, and developing countries (Garvey and Newell, 2005).

This hybrid narrative-systematic literature review sought to provide an overview of the state of evidence for the impacts of CSR on population health. The extent of the literature, that crosses many disciplines and sectors, necessitated a targeted search strategy to yield manageable results for review. This made it difficult to be exhaustive, and there is likely to be other literature the review did not cover that had some bearing on CSR’s impacts on health. We would argue the heterogeneity in the CSR initiatives studied, the research methods used, the quality of studies, and discipline approaches to research we encountered precluded other systematic synthesis methods such as meta-analysis to ascertain an overall conclusion on the effects of CSR on health. However, the narrative approach enables contextualisation of CSR within a broader political economy of health.

## Conclusion

Our review highlights some evidence for potential positive impacts of CSR on health, but a greater range of concerns with the role and intent of CSR that preclude advocating for CSR as a means of improving population health. We would add our call to other authors arguing that meaningful progress on CSR can only be made with greater transparency and reporting of CSR initiatives to allow a fuller evaluation of their impacts (AlFadhli, 2019; Blowfield, 2007). The concerns identified also indicate that it is critical to strengthen government regulation and oversight to maximise any public good that can come from CSR, and minimise the negative consequences reported in research literature. Ultimately, governments are best placed to curtail the health harms of industries, in particular fossil fuels, mining and tobacco, and CSR cannot be seen to allay this urgent need if humanity is to navigate the interrelated poly-crises of climate change, global inequities, and conflict and civil unrest, for all of which corporations are contributing drivers (Jacobs, 2024; Lawrence et al., 2024). Consequently, the need to restrict corporate influence and access to governments – of which CSR is one strategy among many that corporations deploy (Fooks et al., 2013) – remains a vital public health goal. Achieving this goal will require strengthening civil society advocacy and further building political will to legislate strategies such as banning political donations, mandating lobby registers, curtailing the revolving door movement of actors between governments and corporations, and tackling corruption (Wood et al., 2023).
